# Using data mining techniques to explore physicians' therapeutic decisions when clinical guidelines do not provide recommendations: methods and example for type 2 diabetes

**DOI:** 10.1186/1472-6947-9-28

**Published:** 2009-06-10

**Authors:** Massoud Toussi, Jean-Baptiste Lamy, Philippe Le Toumelin, Alain Venot

**Affiliations:** 1 Laboratoire d'Informatique Médicale et Bioinformatique (LIM&BIO EA 3969), UFR SMBH, Université Paris 13, Bobigny, France; 2 Département Inter-hospitalier de Santé publique, Hôpital Avicenne, Assistance publique-Hôpitaux de Paris, Université Paris 13, Bobigny, France

## Abstract

**Background:**

Clinical guidelines carry medical evidence to the point of practice. As evidence is not always available, many guidelines do not provide recommendations for all clinical situations encountered in practice. We propose an approach for identifying knowledge gaps in guidelines and for exploring physicians' therapeutic decisions with data mining techniques to fill these knowledge gaps. We demonstrate our method by an example in the domain of type 2 diabetes.

**Methods:**

We analyzed the French national guidelines for the management of type 2 diabetes to identify clinical conditions that are not covered or those for which the guidelines do not provide recommendations. We extracted patient records corresponding to each clinical condition from a database of type 2 diabetic patients treated at Avicenne University Hospital of Bobigny, France. We explored physicians' prescriptions for each of these profiles using C5.0 decision-tree learning algorithm. We developed decision-trees for different levels of detail of the therapeutic decision, namely the type of treatment, the pharmaco-therapeutic class, the international non proprietary name, and the dose of each medication. We compared the rules generated with those added to the guidelines in a newer version, to examine their similarity.

**Results:**

We extracted 27 rules from the analysis of a database of 463 patient records. Eleven rules were about the choice of the type of treatment and thirteen rules about the choice of the pharmaco-therapeutic class of each drug. For the choice of the international non proprietary name and the dose, we could extract only a few rules because the number of patient records was too low for these factors. The extracted rules showed similarities with those added to the newer version of the guidelines.

**Conclusion:**

Our method showed its usefulness for completing guidelines recommendations with rules learnt automatically from physicians' prescriptions. It could be used during the development of guidelines as a complementary source from practice-based knowledge. It can also be used as an evaluation tool for comparing a physician's therapeutic decisions with those recommended by a given set of clinical guidelines. The example we described showed that physician practice was in some ways ahead of the guideline.

## Background

Clinical guidelines are useful decision support tools for physicians. Their purpose is to bring medical evidence to the point of practice. Physicians need to make clinical decisions based on the available evidence, but they also have to act when such evidence is absent. In many situations, good clinical evidence is impossible, unethical, impractical, or too expensive to generate [1]. As a result, guidelines cannot always provide recommendations for all the possible clinical situations that they are supposed to cover [2]. For example, the French national guidelines for the management of type 2 diabetes mellitus recommends starting oral bitherapy for patients whose HbA1c remains above 6.5% after six months of treatment with maximum dose oral monotherapy [3]. However, it leaves the choice of the appropriate combination of oral anti-diabetic drugs to the physicians' discretion according to their appreciation of the risk/benefit for each patient. This type of situation, where the guidelines "stop" at a certain level and leave the decision to the physician, is common. Unfortunately, it is not always easy for physicians – especially the less experienced – to assess the risk/benefit of each decision in every patient. It would therefore be useful to explore experienced physicians' prescriptions and to analyze how they react in diverse cases with various clinical conditions. This information could then be used to help less experienced physicians with their clinical decision-making.

In our example of type 2 diabetes, the guidelines take a clinical situation into account, but come short of providing full guidance for that situation. Sometimes, guidelines do not take into account a possible patient condition. For example, a former version of the same type 2 diabetes guidelines state that there is no benefit in prescribing an oral tritherapy for a patient whose HbA1c remains above 8% after six months of bitherapy at maximum dose [4]. However, the guidelines do not provide guidance about what to do if such a patient is already under treatment with tritherapy (whether the tritherapy should continue, or be switched to bitherapy, or to insulin therapy). The situation is simply not considered by the guidelines.

Many approaches have been proposed to prevent structural knowledge gaps in guidelines [5]. Some involve guidelines authors following standard structures when developing the guidelines, so as not to leave out any possible patient conditions [6]. Others propose using tools for modeling guidelines such that they can be interpreted by computer, and thus have a more robust logical structure [7]. These approaches help considering possible patient conditions, but they cannot cope with an absence of medical evidence and there are still patient conditions for which modern guidelines do not propose appropriate recommendations [8].

Using the large databases of electronic patient records now available, it is possible to use data mining and knowledge discovery techniques to identify common therapeutic decisions made by physicians for a given clinical condition. There have been some limited attempts at using these techniques for generating practice guidelines from data. Mani et al. [9] presented a two-stage machine learning model as a data mining method to develop clinical practice guidelines, and showed its value in staging dementia. They modeled the methodology used by clinicians by deriving intermediate concepts in the first phase, and in the second phase they used the intermediate concepts for staging dementia. However, the dementia scoring scale that they learnt led to a less complex guideline than those usually implemented in other domains. It is also not clear whether their method can be generalized to different domains. Morik et al. [10] used a combination of prior knowledge from experts and learning from data to generate protocols automatically for decision support in intensive care. They used support vector machines (SVM) to learn the appropriate dose adjustment for each continuously administered drug, based on the response of the patient's vital signs to that change. They then verified the learnt dose adjustment against a medical knowledge base. However, the resulting protocol was again much less complex than most guidelines. In another attempt, Mani et al. [11] used C4.5 and Ripper algorithms with a database of 369 patients and showed that data mining methods could be used for generating simple guidelines and checking compliance to guidelines. Nevertheless, the guidelines resulting from these efforts for creating entire guidelines from data only handle simple problems and lack detail and readability.

Most importantly, guidelines should be evidence-based by definition, and methods aiming at extracting guidelines from data should consider existing evidence-based recommendations. Therefore, another approach can be viewed as filling knowledge gaps of the guidelines rather than replacing them entirely.

Our goal was to develop a method for exploring physicians' therapeutic decisions by use of data mining techniques only in situations for which clinical guidelines do not provide recommendations. We explain the method using the French national guidelines for type 2 diabetes management, and an example database of diabetic patient records. We do not aim at providing medical knowledge from this example.

## Methods

### The guideline

We used the French national guidelines for the management of type 2 diabetes published by the French National Authority for Health (HAS) [4]. A newer version of this guideline was released in 2006 [3]; nevertheless, we analyzed the rules of the previous version, because our patient database was compiled during 2003 and 2004, and also because we wanted to be able to compare the rules extracted by our method with those added to in newer version of the guidelines. The studied guidelines propose a step-by-step treatment strategy for achieving a treatment goal, which is defined by the level of HbA1c. If the goal is not achieved by the ongoing treatment, the physician must step forward to the following types of treatment. The types of treatment consist of diet and exercise, oral monotherapy, oral bitherapy, and insulin therapy alone or with oral anti-diabetic drugs. At each step, the guidelines recommend considering an increase in the dose and then a change in the pharmaco-therapeutic class of the ongoing medication, before advancing to the next step. Many of the recommendations are provided with their level of evidence.

### The patient database

We used a de-identified database of electronic records of ambulatory patients admitted to the Avicenne university hospital in Bobigny, France, for management of their type 2 diabetes, from June 2003 to September 2004. Patients attended the hospital for routine testing of their diabetes or because of its deregulation. They stayed a few hours in the hospital during which time they underwent laboratory tests and consulted a senior staff physician from the department of diabetes care. During the visit, physicians either confirmed the ongoing treatment or changed it. They had access to the guidelines in paper and electronic forms (downloadable .pdf file available at the official HAS website) when entering information into the database. In the database, each patient record contains 125 attributes including administrative (age, sex, consulting physician, physician in charge), anthropometric (weight, height, weight 6 months before, weight at the time of diagnosis, etc), clinical (past or current history of hypertension, coronary artery disease, dyslipidemia, renal insufficiency, cerebrovascular accident, ophthalmopathy, neuropathy, foot problems, smoking, alcohol consumption, level of physical activity, diet, etc), laboratory (HbA1c, morning glycemia, postprandial glycemia, creatinine clearance, microalbuminuria, uricemia, cholesterolemia, etc) and therapeutic (past and current treatments) data. All patient records used in the study were collected for the real practice purposes and not for the study. The retrospectively extracted records were de-identified before being transferred to the authors. As we had no access to the hospital database, nor did we seek any complementary information on patients or doctors (the patient records were completely anonymous), the approval of the study by an ethics committee – beyond the usual declaration made by hospital authorities for the use of patient data – was not necessary.

### Formalizing prescriptions

To facilitate the generation of rules by decision-tree learning algorithms, we formalized the prescriptions by implementing a typology model for drug therapy as described previously [12]. In this model, a drug therapy consists primarily of a type of treatment, which is defined as any combination of drugs (including none) prescribed for a given disease. For example, in type 2 diabetes management, treatment types are classified as diet and exercise, monotherapy, bitherapy, tritherapy, and insulin therapy alone or with oral anti-diabetic drugs. Each type includes a combination of a number (including zero) of drugs having attributes such as pharmaco-therapeutic class, international non-proprietary name (INN), and dose.

### Analyzing the guidelines for missing or incomplete rules

Following Shiffman et al. [13], we formalized recommendations of the guidelines as conditions and actions. We expressed the actions using the typology model for drug therapy described above. We identified all patient variables which were dealt with by the guidelines. We enumerated possible values for each variable and created a table of possible combinations of patient profiles to check whether they were all covered by the guidelines. When possible, we merged some of these profiles into one bigger profile by combining their attributes, in the same as done in the guidelines. For example, in the case of recommendations for metformine prescription, the guidelines merge "renal insufficiency (yes/no)" and "body mass index (BMI) ≥ 28 (yes/no)" into a new attribute of "no renal insufficiency and BMI ≥ 28 (yes/no)" such that only a single (composite) condition has to be verified prior to metformine prescription. In this way, the number of absolute combinations of renal insufficiency and BMI attributes is reduced from 4 to 2.

### Selecting patient records related to missing or incomplete rules

We selected patient records that corresponded to missing or incomplete rules in the guidelines. For example, for patients under treatment with monotherapy with HbA1c above 8%, the guideline recommended bitherapy, without mentioning a preferred combination of pharmaco-therapeutic classes. We extracted all patient records which met this condition from the data base to analyze the preferences of physicians in this group of patients. We proceeded in the same way for all patient profiles corresponding to missing or incomplete rules.

### Generating rules

We used Quinlan's C5.0 decision-tree learning algorithm [14] to generate a set of rules for each subgroup of patient records associated with missing or incomplete rules in the guidelines. We did this in four steps aimed at progressively seeking more detailed rules. First, we implemented the decision-tree learning algorithm to extract rules for the type of treatment for patient profiles for which the guidelines did not provide complete recommendations about treatment type. Then, we extracted rules for the choice of pharmaco-therapeutic class of drugs for patient profiles for which the rules on the choice of the pharmaco-therapeutic class of drugs were missing or incomplete, but where the type of treatment was recommended either by the guidelines or by the rules learnt during the previous step. In the third step, we used the decision-tree learning algorithm to extract rules for the choice of the drug, according to its international non proprietary name (INN), for patient profiles for which the choice of the type of treatment and the pharmaco-therapeutic classes of medications were recommended either by the guidelines or the rules learnt during the previous steps. Finally, we used the learning algorithm to extract rules for the dose of each medication for patient profiles for which the choice of the type of treatment, the pharmaco-therapeutic class and the INN of each drug were recommended by the guidelines or could be calculated using the rules learnt in the previous steps.

### Machine learning methods and options

A C5.0 model works by splitting the sample according to the field that provides the maximum information gain. Each subsample defined by the first split is then split again, usually according to a different field, and the process repeats until the subsamples cannot be split any further. The lowest-level splits are then reexamined, and those that do not contribute significantly to the value of the model are removed or pruned. We used the C5.0 node of the SPSS Clementine^® ^software version 10.1 [15] for machine learning computations.

The C5.0 node can produce two kinds of models: a decision tree which is a straightforward description of the splits found by the algorithm; and a rule set which tries to make predictions for individual records. We chose the first option because it assigns each individual to a single terminal node; whereas an individuals' profile may be associated with more than one rule. Consequently, decision trees are more useful than induced rules for making clinical decisions [16]. To train the C5.0 model, we considered prescription as a dependent variable (*In *field), and all other variables as independent ones (*Out *fields).

We used the Simple Mode option of the C5.0 node which sets most of the C5.0 parameters automatically; these parameters are: pruning severity, minimum records per child branch, global pruning, and Winnow attributes. We set the Favor option of the Simple Mode to generate the most accurate tree possible. We left the Expected Noise option as the default value (0% for the expected proportion of noisy or erroneous data in the training set), because we had thoroughly cleaned our data set prior to use.

We also used the boosting capability of the C5.0 algorithm to improve its accuracy. Boosting works by building multiple models in a sequence. The first model is built in the usual way. A second model is then built in such a way that it focuses especially on the records that were misclassified by the first model. Then a third model is built to focus on the second model's errors, and so on. Finally, cases are classified by applying the entire set of models, using a weighted voting procedure to combine the separate predictions into one overall prediction. This process of boosting can significantly improve the accuracy of a C5.0 model, but it also requires longer training. We set the number of models to be used for the boosting to ten.

Finally, we used the cross validation option of the C5.0 node. It uses a set of models built on subsets of the training data to estimate the accuracy of a model built on the full data set. This is useful if the data set is too small to split into traditional training and testing sets. We set the number of models used for cross validation to ten.

## Results

### Analyzing the guidelines

**Figure 1:**
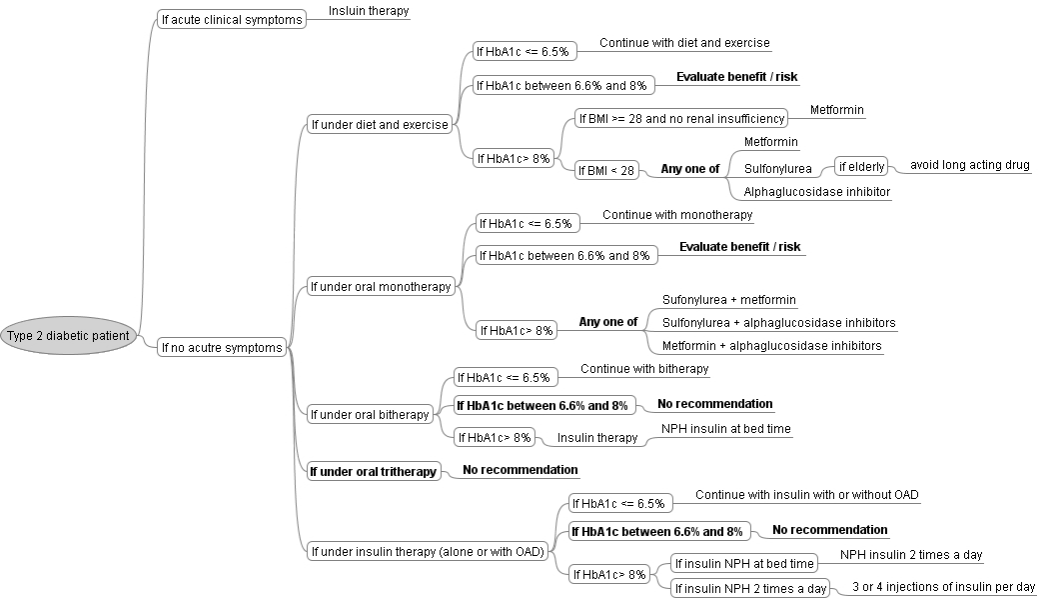
**The decision-tree of the French national guidelines for the management of type 2 diabetes**. Bubbles represent conditions and branches represent actions. Missing or incomplete conditions and actions are shown in bold. OAD: Oral anti-diabetic drugs; Insulin [+OAD]: insulin alone or with oral anti-diabetic drugs.

We analyzed the guidelines by formalizing all the recommendations in conditions and actions, and represented them in a decision-tree (Figure 1).

### Identification of missing or incomplete rules in the guidelines

The guidelines use the following variables to describe patient conditions: acute clinical symptoms (yes or no); HbA1c (three ranges: less than or equal to 6.5%, between 6.6% and 8%, and greater than 8%); type of current treatment (four types: diet and exercise, monotherapy, bitherapy, and insulin therapy alone or with oral anti-diabetic drugs); body mass index (BMI) (two ranges: less than 28, greater than or equal to 28); the existence of renal insufficiency (yes or no); and being old (yes or no). We calculated all combinations of these variables to check if all of them were dealt with by the guidelines. A total of 192 combinations were possible with these variables, but we reduced them to 16 patient profiles (or clinical conditions, designated 1 to 16 hereafter) by merging similar variables, and by using "any" as a denominator when a variable was not dealt with in a particular rule (Table 1).

**Table 1:** Possible patient conditions and their corresponding actions from the guidelines

		Conditions					Actions				
**Profile #**	**Number of patients**	**Acute symptoms**	**HbA1c (%)**	**Ongoing type of treatment**	BMI* (Kg/m^2^)	**Renal insufficiency**	**Elderly**	**Type of treatment**	**Pharmaco-therapeutic class**	**International non proprietary name**	**Dose**

1	2	Yes	Any	Any	Any	Any	Any	Insulin therapy	Insulin	NM***	NM***
2	16	No	≤ 6.5	Any	Any	Any	Any	The same as ongoing treatment	The same as ongoing treatment	The same as ongoing treatment	The same as ongoing treatment
3	7	No	Between 6.6 and 8	Diet and Exercise	Any	Any	Any	Evaluate risk-benefit of continuing or changing the treatment	NM***	NM***	NM***
4	51	No	Between 6.6 and 8	Monotherapy	Any	Any	Any	Evaluate risk-benefit versus of continuing or changing the treatment	NM***	NM***	NM***
5	59	No	Between 6.6 and 8	Bitherapy	Any	Any	Any	NM***	NM***	NM***	NM***
6	16	No	Between 6.6 and 8	Tritherapy	Any	Any	Any	NM***	NM***	NM***	NM***
7	84	No	Between 6.6 and 8	Insulin [+OAD]**	Any	Any	Any	NM***	NM***	NM***	NM***
8	2	No	>8	Diet and Exercise	Any, other than "BMI ≥ 28 and no renal insufficiency"		No	Monotherapy	Any	NM***	NM***
9	1	No	>8	Diet and Exercise	Any, other than "BMI ≥ 28 and no renal insufficiency"		Yes	Monotherapy	Any, other than long acting sulfonylurea	NM***	NM***
10	2	No	>8	Diet and Exercise	≥ 28	No	Any	Monotherapy	Biguanides	Metformine	NM***
11	24	No	>8	Oral monotherapy not at maximum dose	Any	Any	Any	Oral monotherapy	The same as ongoing treatment	NM***	Increase dose until reaching the maximum tolerated
12	1	No	>8	Oral monotherapy at maximum dose	Any	Any	Any	Oral bitherapy	NM***	NM***	NM***
13	39	No	>8	Oral bitherapy with any combination other than "Oral bitherapy with biguanides and sulfonylurea at maximum dose"	Any	Any	Any	Oral bitherapy	Biguanides and sulfonylurea	NM***	NM***
14	18	No	>8	Oral bitherapy with biguanides and sulfonylurea at maximum dose	Any	Any	Any	Insulin [+OAD]**	Insulin [+OAD]**	NM***	NM***
15	18	No	>8	Oral tritherapy	Any	Any	Any	NM***	NM***	NM***	NM***
16	123	No	>8	Insulin [+OAD]**	Any	Any	Any	NM***	NM***	NM***	NM***

* BMI: body mass index

** Insulin [+OAD]: insulin alone or with oral anti-diabetic drugs

*** NM: not mentioned in the guidelines

We distinguished two kinds of knowledge gaps in the guidelines: missing rules and incomplete rules. Missing rules are associated with *conditions *which are not addressed in the guidelines: if a patient condition is not included in the guidelines, then necessarily no corresponding action is proposed. For example, if the condition of patients who come to the doctor under tritherapy is not mentioned in the guideline, the corresponding action to be taken for these patients will also be missing, and the whole rule is therefore missing. Incomplete rules are those related to existing conditions for which the recommended *action *is missing, incomplete or imprecise. Another, better elaborated action is therefore required for an incomplete rule. For example, the condition of patients under monotherapy at maximum dose with HbA1c greater than 8% is considered by the guidelines and a treatment by oral bitherapy is recommended. But the appropriate combination of drugs for such bitherapy is not given by the guidelines. Here, the recommended action does not fully guide the physician to choose the appropriate treatment. The rule is therefore incomplete.

Two physicians and a pharmacist familiar with formalizing guidelines were responsible for the entire process of identifying and classifying of missing and incomplete rules.

### Generating rules

We implemented the C5.0 decision-tree learning algorithm for the type of treatment, the pharmaco-therapeutic class, the INN and the dose of prescribed treatments in 463 patient records divided into 16 groups based on their patient profiles: this process extracted 27 rules (table 2).

**Table 2:** Patient profiles with missing or incomplete rules, and corresponding rules extracted from the database *

Patient subgroups		Learnt rules			
**Profile #**	**Number of patients**	**For type of treatment (instances; confidence rate) [rule precision]**	**For pharmaco- therapeutic class (instances; confidence rate) [rule precision]**	**For INN (instances; confidence rate) [rule precision]**	**For Dose (instances; confidence rate) [rule precision]**

3	7	Diet and exercise (7;1) [100%]	Not applicable		
4	51	Monotherapy (45;1) [100%]	sulfonylurea if dyslipidemia (7;0.89), else alpha glucosidase inhibitors if not already under biguanides (3;0.8); biguanides by default (11;0.92) [100%]		
5	59	Bitherapy (56;1) [100%]	Biguanides and sulfonylurea [100%]	Metformine and gibenclamide [100%]	
6	16	Insulin [+OAD]** if cholesterolemia ≤ 400 mg/dl (2;0.75), Tritherapy if not (12;0.93) [100%]	Biguanides, sulfonylurea and alpha glucosidase inhibitors if tritherapy [100%]		
7	84	Insulin [+OAD]** (75;1) [100%]	Insulin only if already under insulin plus biguanides (62;0.65); Insulin with biguanides if postprandial glycemia >184 mg/dl and age<52 yrs (11;0.92); Insulin with sulfonylurea and alpha glucosidase inhibitors if already under sulfonylurea (11;0.63) [100%]		
12	1	Oral bitherapy (mentioned in the guideline)	Biguanides and sulfonylurea [100%]		
13	39	Oral bitherapy (mentioned in the guideline)	Biguanides and sulfonylurea if bitherapy [100%]		
14	18	Insulin [+OAD]** (mentioned in the guideline)			
15	18	Insulin [+OAD]** if morning glycemia >230 mg/dl (8;0.8), tritherapy if not (10;0.92) [100%]	Biguanides, sulfonylurea and alpha glucosidase inhibitors if tritherapy [100%]		
16	123	Insulin [+OAD]** (114;1) [100%]	Insulin only if already under insulin plus sulfonylurea or insulin only (72;0.78); Insulin plus biguanides if already under insulin plus biguanides (24.5;0.83); insulin plus biguanides and sulfonylurea if not (17;0.36) [90.9%]		One injection of Insulin if accompanied by biguanides or sulfonylurea (10;0.83); Two injections of insulin [+OAD]** (104;0.79) [72.7%]

* Rules are separated according to the typology of treatment: the level of detail increases from left to right. Following each rule, its number of instances and confidence rate are presented in parentheses and the rule precision is given in square brackets.

** Insulin [+OAD]: Insulin alone or with oral anti-diabetic drugs.

The rules generated by the C5.0 algorithm are presented in the following format:

if antecedent_1 and antecedent_2 ... and antecedent_n

then consequent

where *consequent *and *antecedent_1 *through *antecedent_n *are all variables. The rule is interpreted as "for records where *antecedent_1 *through *antecedent_n *are all true, *consequent *is also likely to be true." Information on the number of records to which each rule applies – that is, for which the antecedents are true (Instances) – and the proportion of those records for which the entire rule is true (Confidence) is given in parenthesis for each rule in Table 2. The proportion of the number of true positive records to that of true positive and false positive records classified by the rules (Precision) for each model is also reported in square brackets. For example, in the last row of Table 2, for the profile number 16 which comprised 123 cases, the algorithm found a rule for the type of treatment in 114 records (Instances): it proposed prescribing insulin alone or with anti-diabetic drugs. The rule was true for all of 114 instances, i.e. both conditions and the action were true for all of these records (Confidence: 1.0). Furthermore, the proportion of true positive records to the total number of true positive and false positive records (Precision) was 100% for the model (which means that there were no false positive records for this model). The algorithm could not however find any rules for the remaining 9 (= 123-114) records in this profile.

When the same action was applied to all patients of a sample without considering any condition, the result was only a default rule with its precision. A default rule is a rule that represents a non-conditional action. For example, we applied the algorithm for learning the pharmaco-therapeutic class of drugs in a group of ten patients for whom the C5.0 model had already proposed tritherapy in a previous step (see the next-to-last line of Table 2). The fourth column of Table 2 shows that all of the ten patients were treated with biguanides, sufonylurea, and alpha glucosidase inhibitors. As there was no other combination of pharmaco-therapeutic classes to learn, the algorithm cannot produce a real tree (or it can produce a tree with only one branch), which we call a default rule.

### Rules pertaining to the choice of the type of treatment

We used the C5.0 decision-tree learning algorithm for every group of patients with profiles for which the type of treatment was missing, incomplete or imprecise (profile numbers 3 – 7, and 15 – 16, see Table 1). We considered the type of treatment as a dependent variable, and all other relevant variables as independent. The algorithm extracted 11 rules from physicians' prescriptions for some of these profiles (Table 2). For example, it revealed that for patients who were already under tritherapy and had HbA1c > 8% (profile 15), physicians prescribed insulin therapy with or without oral anti-diabetic drugs if morning glycemia was greater than 230 mg/dl; if it was lower, they continued tritherapy.

### Rules pertaining to the choice of the pharmaco-therapeutic class of drugs

We considered patient profiles for which the type of treatment was known (either through the guidelines or from the previous step) but the pharmaco-therapeutic class of medications was missing or incomplete or imprecise (profile numbers 3 – 7, 9, 12, and 14 – 16). We used the C5.0 decision-tree learning algorithm to explore physicians' choices about the pharmaco-therapeutic classes of drugs for these cases (Table 2). In creating the model, the pharmaco-therapeutic class of drugs was considered as a dependent variable and all other relevant variables (including the type of treatment) as independent. Thirteen rules were extracted concerning pharmaco-therapeutic classes of medications. For example, the algorithm learnt that for all patients treated with maximum dose oral monotherapy and having HbA1c > 8% (profile 12), physicians prescribed a combination of biguanides and sulfonylurea.

### Rules pertaining to the choice of drugs (international non proprietary name) and doses

We considered patient profiles for which the type of treatment and the pharmaco-therapeutic classes of drugs were already known either directly from the guidelines or from previous steps (profiles numbered 4 – 9). We used C5.0 decision-tree learning algorithm to extract rules related to drug choice (according to their INNs). In a following step, we used the C5.0 learning algorithm to extract rules for the dose of drugs in profiles for which all other elements of treatment (namely, type, pharmaco-therapeutic class, and drug INN) were already known (profiles 11 – 16). The extraction of rules was less successful in these steps because the conditions and profiles had been already multiplied in the previous steps (Table 2), and therefore, most of groups of patients did not contain enough records to allow the algorithm to calculate rules. However, we were able to extract one rule for the drug choice (according to its INN) and two rules for drug doses (5^th ^and 6^th ^columns of table 2). For example, for all patients receiving bitherapy with HbA1c between 6.6 and 8% (profile 5), and for whom the learning algorithm had proposed bitherapy with biguanides and sulfonylurea (56 patients out of 59), the extracted rule suggested to prescribe invariably metformine and glibenclamide. Also, for patients with HbA1c above 8% (profile 16), receiving insulin alone or with oral anti-diabetic drugs, the extracted rule suggested one daily insulin injection if the patient received also biguanides and sulfonylurea (Incidence:10 patients), but it suggested two daily insulin injections if the patient received another combination of oral anti-diabetic drugs with the insulin (Incidence:104 patients).

### Grafting the rules learnt onto the decision-tree of the guidelines

**Figure 2:**
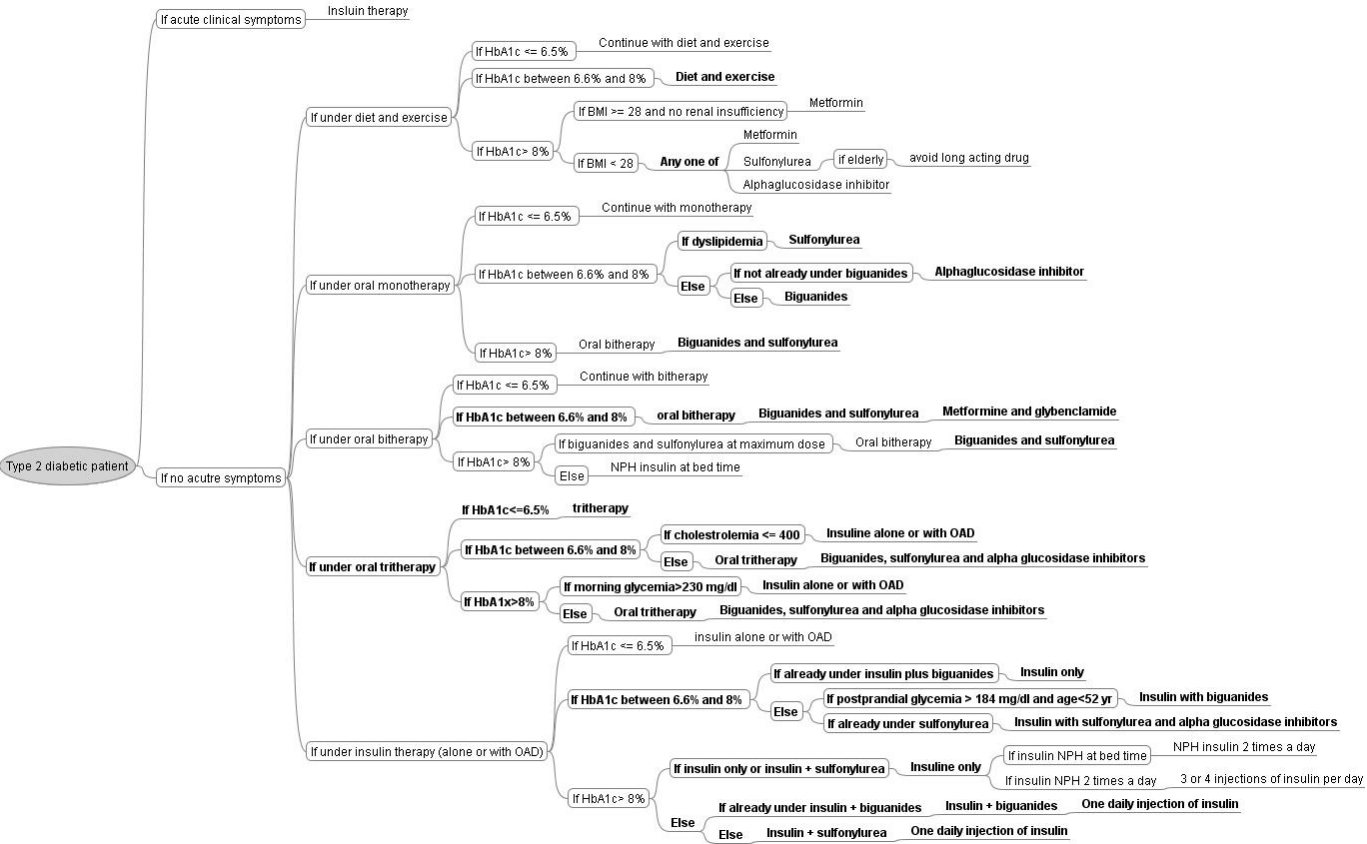
**The decision-tree of the original guideline with the learnt rules grafted on it**. Bubbles represent conditions and branches represent actions. The grafted rules are shown in bold. OAD: Oral anti-diabetic drugs; Insulin [+OAD]: insulin alone or with oral anti-diabetic drugs.

We grafted the rules learnt onto the decision-tree of the guidelines (figure 2) to assess the readability and understandability of the new rules. The resulting grafted decision-tree covers more clinical situations – especially with regard to tritherapy and insulin therapy – and provides much more detailed and elaborated recommendations than the decision-tree of the original guidelines. Note that the original guidelines had few rules for insulin therapy, whereas the grafted guidelines are much more explicit in this regard.

### Comparison with the new version of the guideline

We tried to compare the extracted rules with the new rules in the updated version of the guidelines to identify similarities. However, the new guidelines were fundamentally different from the original, for example in providing different HbA1c thresholds for each type of treatment. Consequently, it was difficult to make comparisons. In this work, we expressly did not seek to learn rules to replace existing evidence-based rules in the original guidelines. As a result, the grafted decision-tree retained the basic structure of the original guidelines, which was different from that of the new version. A one-by-one comparison of our rules with the newer guideline was therefore not possible. Nevertheless, we observed some similarities. One major structural similarity was the recommendation of oral tritherapy for patients under bi- or tritherapy. The original guidelines did not acknowledge the usefulness of oral tritherapy in the treatment of type 2 diabetes. We learnt from the database that tritherapy was prescribed by physicians under some circumstances during 2003 and 2004, and this in spite of their contemporary guidelines. The usefulness of tritherapy was confirmed by the new version of the guidelines in 2006.

### A tree entirely based on physicians' decisions

**Figure 3:**
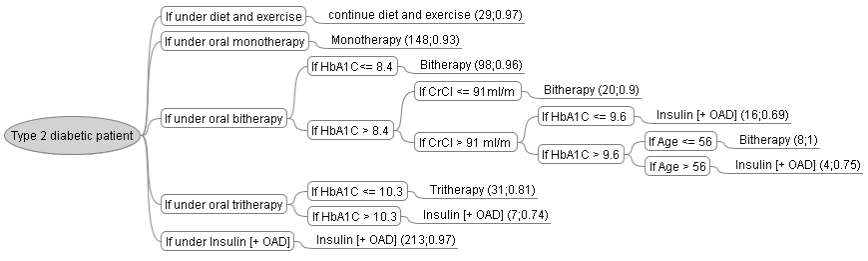
**Reconstruction of physicians' decision-tree using the C5.0 learning algorithm with instances and confidence rate for each rule in parentheses**. Only the rules leading to the choice of the type of treatment are represented. CrCl: creatinine clearance; OAD: oral anti-diabetic drugs.

Out of intellectual curiosity, we constructed the decision-tree corresponding to physicians' decisions, and compared it to the original and the new guidelines. We applied the C5.0 decision-tree learning algorithm to the entire database to extract rules that explained physicians' prescriptions on the basis of patient variables. We filtered all patient variables except for those used by the guidelines to force our algorithm to generate rules which could be comparable with those of the guidelines (Figure 3).

Physicians -like the new guidelines- used multiple thresholds of HbA1c for each type of treatment (Figure 3), but their thresholds were generally higher than those used by the guidelines (8.4%, 9.6% and 10.3% versus 6%, 6.5% and 8%). Physicians, again like the guidelines, used BMI for decisions concerning the pharmaco-therapeutic class and the choice of drugs (INN). The treatment types however were dependent only on the current types, and physicians did not use other patient conditions when making these decisions. This learnt algorithm appears to be more naive than that could be constructed in combination with the guidelines.

## Discussion and conclusion

We used an example to demonstrate how to explore the knowledge gaps in guidelines, and how to use data mining techniques to extract rules from patient records to provide supplementary information for these knowledge gaps. The extracted rules permitted us to complete the decision-tree of the guidelines studied up to the level of pharmaco-therapeutic class of medications for all patient profiles, and up to the level of drug choice (according to the INN) and dose for some profiles. With a database containing more patient records, it would be probably possible to fill in all gaps of the guidelines up to the level of dose for all patient profiles.

As we make use of general concepts of the guidelines and not their disease-specific characteristics, our approach may be applied with little change to other therapeutic guidelines at least in the domain of chronic diseases.

We have already proposed a method using a decision-tree learning algorithm for the evaluation of a guideline-based decision support system [17]. The current approach we propose is however different from that of our previous study, because in this study we aimed at exploring physicians' prescriptions rather than evaluating the results of a system, and consequently, we applied the decision-tree learning algorithm to selected groups of patient records rather than to the whole database.

The use of machine learning techniques for creating guidelines from data has been suggested previously [9-11,18-20]. However, we proposed a new our approach, using the data mining for enriching the guidelines and not for creating them from scratch.

Although the validity of the rules extracted in our approach depends on the quality of prescriptions and the size of the database; the validity of the method itself is not dependent of these elements.

We showed that some of the decision rules extracted from physicians' prescriptions were similar to those added to the new version of the guidelines released three years after the database was being filled. This suggests that the therapeutic reasoning of experienced physicians was in some ways ahead of their contemporary guidelines, i.e. they could "anticipate" the guidelines update. This is presumably because guidelines are based on medical evidence, and their development is time consuming; consequently, the results of medical progress appear in guidelines only after a delay, whereas physicians' practice is generally influenced by the most recently published articles [21].

Although not initially among our objectives, we constructed the whole decision-tree corresponding to physicians' decisions by using the algorithm with the entire database. This revealed that some aspects of physicians' therapeutic decisions (for example the usefulness of tritherapy, or the use of different HbA1c thresholds for different types of treatment) were similar to the newer version of the guidelines. However, we did not intend to infer a complete set of guidelines. We do believe that guidelines must be as evidence-based as possible, and the evidence must come from scientific studies (clinical trials, etc). As a result, the approach proposed in this article can be considered useful when appropriate scientific evidence does not exist but data corresponding to the opinion of leading experts – whose practice can be considered as standard or optimal for the medical issues considered – are available.

We used the C5.0 decision-tree learning algorithm. C5.0 models are robust in the presence of problems such as missing data and large numbers of input fields. They tend to be easier to understand than some other model types, because the interpretation of the rules derived from the model is very straightforward. C5.0 models also allow the use of a powerful boosting method for increasing the accuracy of classification.

A common potential limitation of decision-tree learning algorithms is overfitting [22]. This occurs when the decision-tree characterizes too much detail, leaving no place for fitting future cases. The C5.0 decision-tree learning algorithm is probably less susceptible to this problem than its predecessor, C4.5, because it generates smaller decision-trees. The automatic pruning of the Simple Mode of C5.0 also reduces overfitting [15].

Our method can be used as a simple and quick way to generate practice-based decision-rules for knowledge gaps in guidelines. For example, the authors and editors of guidelines could use our method as a support for discussing "expert opinion" parts of their guidelines. This is especially useful as supportive approach to the laborious process of adopting national or international guidelines for local use [23]. It can also be used to facilitate the implementation of guidelines in decision support systems by providing practice-based rules to complete their logical structure.

## Abbreviations

HAS: Haute autorité de santé; OAD: oral anti-diabetic drugs; CrCl: creatinine clearance; BMI: body mass index; INN: International non proprietary name; NM: not mentioned in the guidelines; Insulin [+OAD]: insulin alone or with oral anti-diabetic drugs.

## Competing interests

The authors declare that they have no competing interests.

## Authors' contributions

MT carried out computer methods, participated in data collection and preparation, and drafted the manuscript. JBL contributed to the methods and to writing the manuscript. PLT provided the database and participated in the preparation and selection of patients in the database. AV supervised the study, and participated in its design and coordination and helped to draft the manuscript. All authors read and approved the final manuscript.

## Pre-publication history

The pre-publication history for this paper can be accessed here:

http://www.biomedcentral.com/1472-6947/9/28/prepub
